# HMGB1/PI3K/Akt/mTOR Signaling Participates in the Pathological Process of Acute Lung Injury by Regulating the Maturation and Function of Dendritic Cells

**DOI:** 10.3389/fimmu.2020.01104

**Published:** 2020-06-19

**Authors:** Ruiting Li, Xiaojing Zou, Haiyan Huang, Yuan Yu, Hongmei Zhang, Pei Liu, Shangwen Pan, Yaqi Ouyang, You Shang

**Affiliations:** ^1^Department of Critical Care Medicine, Institute of Anesthesia and Critical Care Medicine, Union Hospital, Tongji Medical College, Huazhong University of Science and Technology, Wuhan, China; ^2^Department of Emergency, Shenzhen People's Hospital, First Affiliated Hospital of Southern University of Science and Technology, Second Clinical Medical College of Jinan University, Shenzhen, China; ^3^Shenzhen Key Laboratory of Respiratory Diseases, Department of Respiratory and Critical Care Medicine, Shenzhen Institute of Respiratory Diseases, Shenzhen People's Hospital, First Affiliated Hospital of Southern University of Science and Technology, Second Clinical Medical College of Jinan University, Shenzhen, China

**Keywords:** HMGB1, PI3K/Akt/mTOR signaling, acute lung injury, lipopolysaccharide, dendritic cell

## Abstract

**Background:** High-mobility group box 1 protein (HMGB1) was identified as a highly conserved DNA binding nuclear protein, which participates in the processes of acute lung injury (ALI). HMGB1 binds to its specific receptors not only to activate the nuclear factor (NF)-κB and mitogen-activated protein kinase (MAPK) pathways but also to regulate the activation of the phosphatidylinositol 3′-kinase/protein kinase B/mammalian target of the rapamycin (PI3K/AKT/mTOR) pathway. Mature dendritic cells (DCs) regulate acute lung inflammation and pathological injury in ALI. In addition, studies have shown that the activation of the PI3K/AKT/mTOR signaling pathway may regulate the function and maturation of DCs.

**Objective:** Therefore, we speculate that HMGB1/PI3K/Akt/mTOR signaling participates in regulating the pathological process of ALI by regulating the maturation and function of DCs.

**Methods:** Anti-HMGB1 antibody, rHMGB1, or LY294002 (PI3K inhibitor) was administered in a murine model of lipopolysaccharide (LPS)-induced ALI. For *in vitro* studies, generated bone marrow-derived dendritic cells (BMDCs) primed by LPS were stimulated with the same reagents. The effects of these different treatments were observed on the expression of PI3K, AKT, and mTOR and on the function of DCs.

**Results:** HMGB1 upregulated the expression of PI3K, Akt, and mTOR mRNA and phosphorylated proteins in BMDCs. The HMGB1/PI3K/Akt/mTOR signaling pathway induced the maturation and antigen-presenting ability of lung DCs, mediated the percentage of myeloid DCs (mDCs), and enhanced the adhesion and chemotactic ability of lung DCs.

**Conclusions:** HMGB1/PI3K/Akt/mTOR signaling participates in the pathological process of ALI by regulating the maturation and functions of DCs.

## Introduction

Acute lung injury (ALI)/acute respiratory distress syndrome (ARDS) is a complex clinical syndrome characterized by persistent hypoxemia due to pulmonary interstitial edema, damage and disruption of the alveolar–capillary barrier, and widespread inflammation in the lung (1, 2). Although the clinical treatment and management of ALI/ARDS have improved, it still has high morbidity and mortality in the intensive care unit (3). Previous studies have shown that an excessive inflammatory response storm plays a vital role in the pathological process of ALI/ARDS (1). Therefore, elucidation of molecular and cellular mechanisms associated with inflammation in ALI may be helpful in identifying new therapeutic targets.

High-mobility group box 1 protein (HMGB1) was identified as a highly conserved DNA binding nuclear protein and participates in the processes of replication, recombination, transcription, and DNA repair. HMGB1 is reported to contribute to inflammatory dysfunction in sepsis and ALI, and the levels of HMGB1 in the plasma and tissue were significantly increased in a mouse model of lipopolysaccharide (LPS)-induced mouse model of ALI (4, 5). Increasing evidence supports the role of HMGB1 as a regulator of ALI. The downstream pathways of HMGB1 may lead to neutrophil infiltration, injury of lung tissue, inflammatory cytokine release, and the development of ALI. HMGB1 binds to its specific receptors, including the receptor for advanced glycation end products, toll-like receptor (TLR)2 and TLR4, in turn activating the nuclear factor (NF)-κB and mitogen-activated protein kinase (MAPK) pathways, which mediate inflammatory molecules such as TNF-α, IL-1β, IL-18, and IL-6 (6–8). HMGB1 also induces an inflammatory response through the phosphatidylinositol 3′-kinase/protein kinase B/mammalian target of the rapamycin (PI3K/AKT/mTOR) pathway (9). Previous studies have shown that HMGB1 inhibitors or HMGB1 siRNA effectively inhibited the activation of the TLR4/NF-κB and PI3K/AKT/mTOR pathways (9, 10). Previous research has shown that the PI3K/Akt/mTOR pathway regulated multiple physiological activities such as cell proliferation, autophagy, and apoptosis (11, 12). In addition, the PI3K/Akt/mTOR pathway has an important role in pulmonary inflammation and pathological progression of ALI (13, 14). Thus, HMGB1 induces an inflammatory response in ALI through the PI3K/AKT/mTOR pathway.

Dendritic cells (DCs), the most prominent antigen-presenting cells, play a key role in initial and adaptive immune responses and are ideally positioned to serve a priming and central role in the immune response during inflammation (15, 16). Previous studies have shown that the number of mature conventional DCs (cDCs) in lung tissue during ALI is significantly increased, and the maturation of pulmonary DCs regulate acute lung inflammation and pathological injury (17, 18). Therefore, DCs play an important role in the pathological progress of ALI, and regulating the function and maturation of DCs may have great clinical significance for the treatment of ALI.

Studies have shown that promoting the activation of PI3K/AKT/mTOR signaling regulates the function of immune cells including DCs (19, 20). Moreover, the activation of the PI3K/Akt/mTOR pathway participates in sepsis-induced ALI (11, 13, 21). Thus, we speculated that HMGB1/PI3K/Akt/mTOR signaling participates in regulating the pathological process of ALI by regulating the maturation and function of DCs.

## Materials and Methods

### Mice

Male C57BL/6 mice (6–8 weeks old, 20–22 g) were obtained from the Experimental Animal Centre of Hubei province (Wuhan, China). The mice were maintained in the animal laboratory of the Experimental Animal Center of Hubei province under standard laboratory conditions for 1 week prior to the experiments. The mice were kept under specific pathogen-free conditions at 25°C in a 12-h light/dark cycle, with a humidity of 45–55% in a ventilated cage. All experimental procedures were performed in accordance with the requirements of the Institutional Animal Care and Use Committee at Huazhong University of Science and Technology (Wuhan, China).

### Experimental Protocol for the ALI Murine Model

All mice were divided into the following groups using the randomized grouping method (*n* = 4–6 per group): control group, LPS group, LPS+anti-HMGB1 group, LPS+rHMGB1 group, LY294002 (PI3K inhibitor) positive control group, and LPS+LY294002 group. The ALI murine model was induced by intraperitoneal (i.p.) injection of LPS as described previously (22). Anti-HMGB1 or rHMGB1 was administered as previously described (22). The LY294002 intervention group was injected with 1 mg/25 g LY294002 via the tail vein 2 h after LPS injection (23). All experimental mice were sacrificed using cervical dislocation 24 h after receiving the LPS challenge, and lung tissues and bronchoalveolar lavage fluid (BALF) were extracted for further analysis.

### Collection of BALF

We collected BALF as described in detail in a previous study (24).

### Generation of Bone Marrow-Derived Dendritic Cells (BMDCs)

BMDCs were generated as previously described (24). The obtained BMDCs were stimulated with or without 1 μg/ml LPS (Sigma Aldrich, St. Louis, MO, USA), anti-HMGB1 (10 μg/ml) (22), rHMGB1 (50 μg/ml) (22), or LY294002 (25 μM) (25) for 24 h. BMDCs and cell supernatants were collected for subsequent real-time reverse-transcriptase polymerase chain reaction (RT-qPCR) and enzyme-linked immunosorbent assay (ELISA) analyses.

### Cytokine Analysis

Concentrations of interleukin (IL)-12p40, tumor necrosis factor (TNF)-α, IL-6, IL-18, IL-1β, and monocyte chemotactic protein (MCP)-1 secreted by DCs from each sample were determined by ELISA according to the manufacturer's instructions (eBioscience, San Diego, CA, USA). Cytokine concentrations are expressed as pg/ml.

### RNA Extraction and RT-qPCR

Total RNA was extracted from the right lung tissue and BMDCs by a Trizol reagent (Invitrogen/Thermo Fisher Scientific Inc., Carlsbad, CA, USA), and the RNA was reverse-transcribed into complementary DNA (cDNA) using a ReverTra Ace qPCR RT kit (Toyobo CO., LTD., Tokyo, Japan) according to the manufacturer's protocol.

RT-qPCR assay on the samples was carried out using the SYBR Premix Ex Taq™ (Takara Bio Inc., Otsu, Japan) as previously described (24, 26). RT-qPCR data were analyzed by QuantStudio 6 Flex (ABI Life Technology, USA). The 2^Δ*ΔCt*^ method was used to evaluate the relative expression of each target gene after normalization by glyceraldehyde 3-phosphate dehydrogenase (GAPDH). Primer sequences for each target gene are listed in Table 1.

**Table 1:** Primer sequences and product sizes.

**Gene name**	**Primer sequences (5^**′**^-3^**′**^)**	**Product size (bp)**
PI3K Forward:Reversed:	5′- ACACCACGGTTTGGACTATGG -3′ 5′- GGCTACAGTAGTGGGCTTGG -3′	140
Akt Forward:Reversed:	5′- TGGGTCAAGGAACAGAAGCA -3′ 5′- TCACACTGACCACTGACACA -3′	111
mTOR Forward:Reversed:	5′-CGGGACTCTTTACACTGCG-3′ 5′-CCTTCAGGCTCAACCAACA-3′	82
GAPDH Forward:Reversed:	5′-TGTGTCCGTCGTGGATCTGA-3′ 5′-TTGCTGTTGAAGTCGCAGGAG-3′	150

*(a) PI3K, Phosphatidylinositol 3′-kinase; (b) Akt, Protein kinase B; (c) mTOR, Mammalian target of rapamycin; (d) GAPDH, Glyceraldehyde-3-phosphate dehydrogenase*.

### Western Blot Analysis

BMDCs were harvested at 24 h after stimulation with LPS and frozen at −80°C. Total protein was extracted using a lysis buffer (Beyotime Institute of Biotechnology, Haimen, China). Protein concentrations were determined by BCA Protein Assay Kit (Beyotime Biotechnology, Shanghai, China). Protein (40 μg/well) was separated via 10% sodium dodecyl sulfate-polyacrylamide gel electrophoresis (SDS-PAGE) and transferred to polyvinylidene fluoride (PVDF) membranes (Millipore Corp., Billerica, MA, USA). Membranes were blocked with 5% non-fat milk in Tris-buffered saline containing Tween 20 (TBST) for 2 h at room temperature, then incubated with primary antibodies (p-PI3K p85, p-Akt T308, p-mTOR, Abcam, Cambridge, UK) diluted in a blocking solution (1:1,000) overnight at 4°C, and finally treated with diluted horseradish peroxidase (HRP)-conjugated secondary antibody (1:50,000, BOSTER Biological Technology Co., Wuhan, Chine) at 37°C for 2 h. The immunoreactive bands were analyzed using the BandScan 5.0 (Glyko, Novato, CA, USA) gel imaging software.

### Flow Cytometry

To determine the phenotype of DCs in lung tissue and BMDCs, prepared lung mononuclear cells (MNCs) and BMDCs were suspended in a FACS buffer at 2 × 10^6^ cells/ml. DCs express the marker CD11c but not the marker F4/80 on the cell surface; therefore, DCs were gated as CD11c^+^/F4/80^−^ cells using the PE-CD11c antibody (eBioscience, San Diego, CA, USA) and the APC-Cy7-F4/80 antibody (BioLegend Inc., San Diego, CA, USA). Next, the cells were stained with the following antibodies: FITC-MHCII, FITC-CD80, FITC-CD40, FITC-B220, FITCICAM-1 (eBioscience, San Diego, CA, USA), APC-CD86, APC-CCR7, APC-CD11b (Biolegend Inc., San Diego, CA, USA). All cells were analyzed by flow cytometry (FACSAria™ III, BD Biosciences, USA).

### Statistical Analysis

The data are presented as the mean ± standard deviation (SD). All experiments were repeated at least three times. One-way analysis of variance was used to compare differences. Statistical analysis was carried out using the GraphPad Prism 6 software (GraphPad Software Inc., San Diego, CA, USA) and the SPSS 22.0 software (IBM SPSS, Chicago, IL, USA). Differences were considered significant at *p* < 0.05. ^*^ *p* < 0.05; ^**^ *p* < 0.01; and ^***^ *p* < 0.001.

## Results

### HMGB1 Activated PI3K/Akt/mTOR Signal Pathway in BMDCs

**Figure 1:**
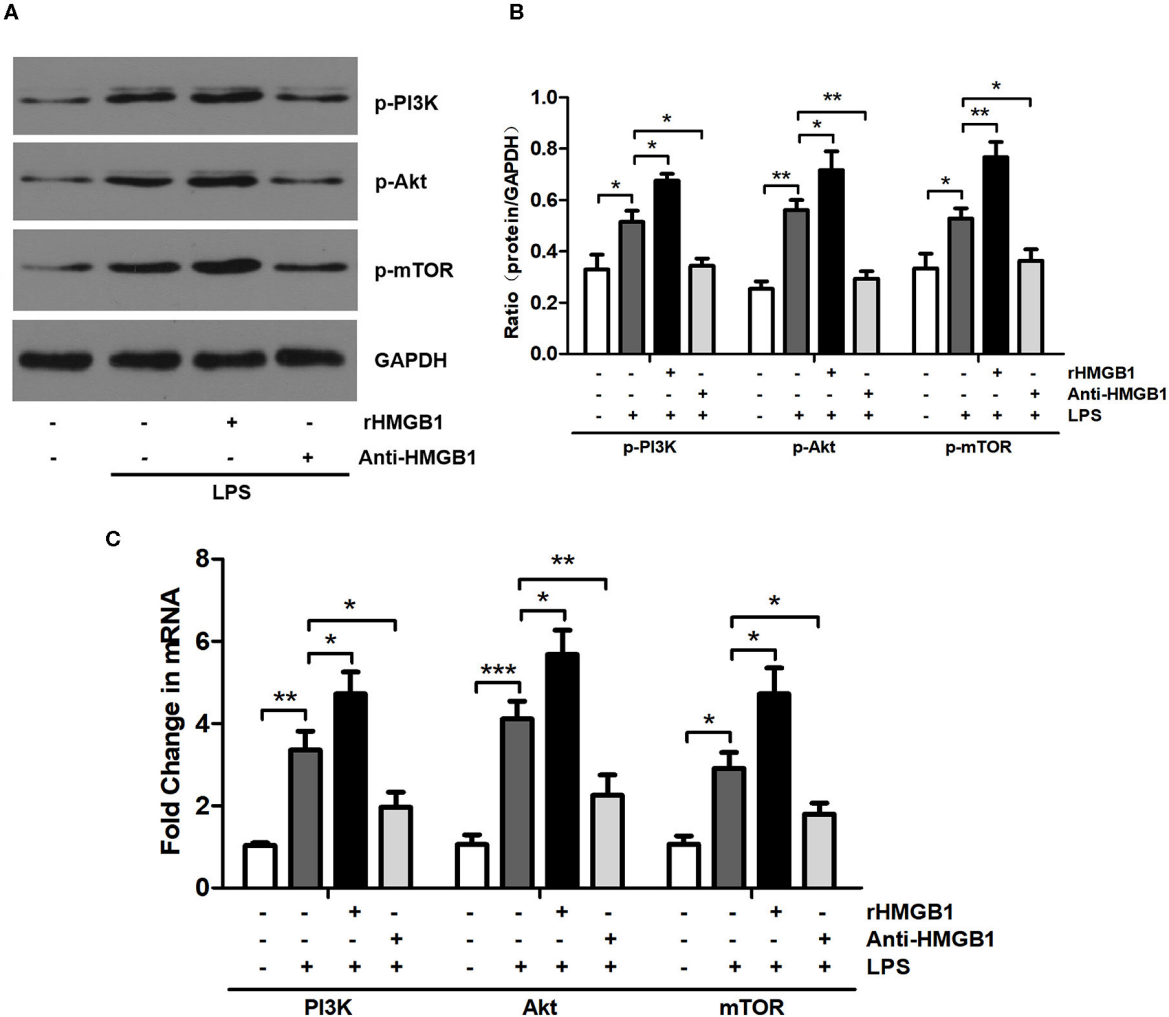
Anti-HMGB1 and rHMGB1 regulated the expression of the PI3K/Akt/mTOR signaling pathway in BMDCs. **(A,B)** Expression of p-PI3K, p-Akt, and p-mTOR in BMDCs of different groups was measured by Western blot analysis. GAPDH served as a loading control. **(C)** Expression of PI3K, Akt, and mTOR mRNA in BMDCs of different groups was measured by RT-PCR. GAPDH served as the housekeeping gene. *p < 0.05, **p < 0.01, ***p < 0.001.

Previous studies have shown that HMGB1 regulates the PI3K/Akt/mTOR pathway in myocardial ischemia reperfusion injury and the ALI mouse model (9, 27). We examined the effect of HMGB1 on PI3K/Akt/mTOR signaling in BMDCs following administration with rHMGB1 or anti-HMGB1 by Western blot and RT-qPCR analysis. HMGB1-mediated PI3K/Akt/mTOR pathway activation was assessed by detecting p-PI3K, p-Akt, and p-mTOR protein and PI3K, Akt, and mTOR mRNA expression levels. Western blot and RT-qPCR analysis revealed a remarkable increase in the expression of p-PI3K, p-Akt, and p-mTOR protein (Figures 1A,B) and PI3K, Akt, and mTOR mRNA (Figure 1C) in LPS-primed BMDCs. Moreover, rHMGB1 treatment further increased the expression of these proteins and transcripts, but this increase was attenuated by anti-HMGB1 (Figures 1A–C). These results suggest that HMGB1 is an activator of the PI3K/Akt/mTOR pathway in DCs.

### HMGB1 Induced the Maturation of DCs *in vivo* and *in vitro*

**Figure 2:**
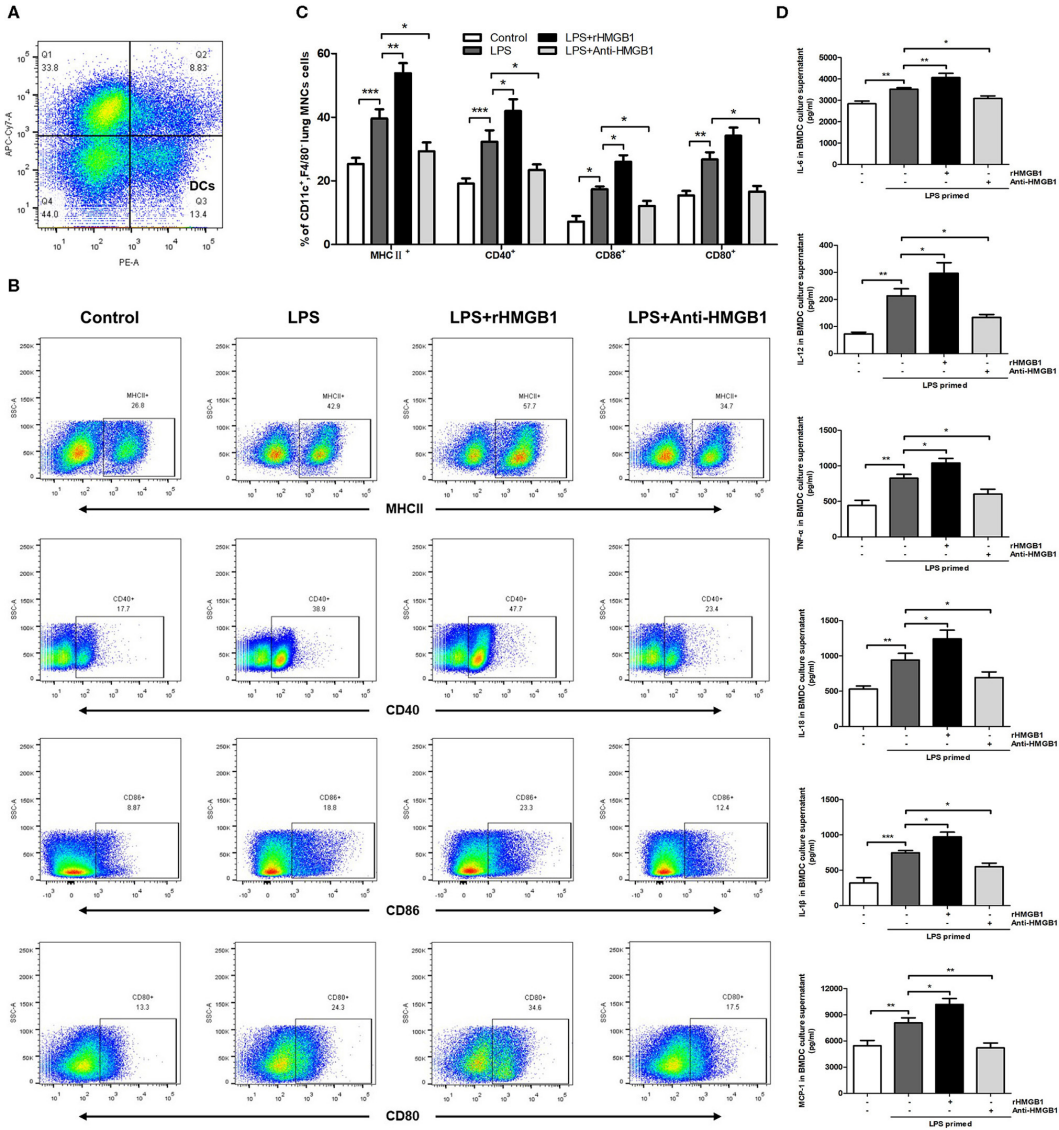
Anti-HMGB1 and rHMGB1 regulated the maturation of DCs *in vivo* and *in vitro*. **(A)** Lung DCs were stained as CD11c^+^F4/80^−^. **(B,C)** Positive expression percentage of MHCII, CD80, CD86, and CD40 was measured in DCs (CD11c^+^F4/80^−^) by flow cytometric analysis. **(D)** The levels of cytokines TNF-α, IL-6, IL-18, IL-1β, MCP-1, and IL-12 secretion in BMDC culture supernatants were measured by ELISA. *p < 0.05, **p < 0.01, ***p < 0.001.

The activation and maturation of DCs are characterized by the expression of MHCII and various costimulatory molecules on their surface and the secretion of related inflammatory cytokines (28, 29). To determine whether HMGB1 affects the maturation and function of DCs, we observed the effect of HMGB1 on MHCII, CD80, CD86, and CD40 of lung DCs in the LPS-induced ALI model. The left lungs from mice of different groups were collected 24 h after LPS intervention, and the prepared lung MNCs were stained for CD11c and F4/80 and analyzed by flow cytometry. DCs were marked as CD11c^+^F4/80^−^ (Figure 2A). The analysis results showed that the positive expression percentage of MHCII, CD80, CD86, and CD40 was significantly increased in the ALI group in contrast to those in the control, and the increase in MHCII, CD86, and CD40 was further augmented by rHMGB1 treatment. By contrast, the increase in MHCII, CD80, CD86, and CD40 was obviously lowered in the ALI group treated with anti-HMGB1 (Figures 2B,C). Subsequently, we observed the effect of HMGB1 on proinflammatory cytokines released by LPS-primed BMDCs *in vitro*. The levels of TNF-α, IL-6, IL-18, IL-1β, MCP-1, and IL-12 cytokines released by DCs, which reflect the maturation of DCs, were significantly upregulated by LPS stimulation in the BMDC culture supernatant compared with those in the control group. On the other hand, rHMGB1 stimulation also significantly increased the production of these proinflammatory cytokines. Opposite results were observed in the groups receiving anti-HMGB1 stimulation (Figure 2D). These data suggested that HMGB1 induces the maturation and enhances the antigen-presenting ability of DCs in the ALI mice model and in LPS-primed BMDCs.

### HMGB1 Affected the Phenotypic Changes of DCs in ALI Mice Model

**Figure 3:**
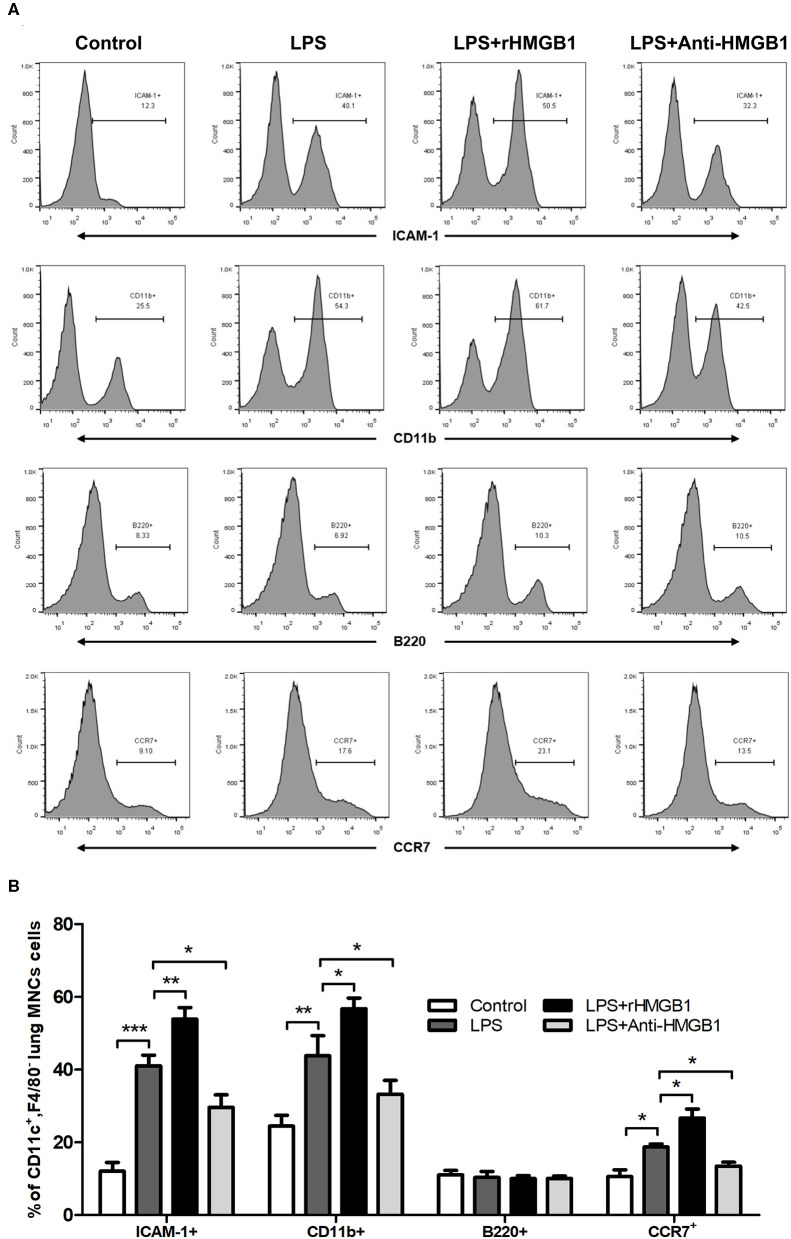
Anti-HMGB1 and rHMGB1 affected the phenotype and function of DCs. **(A,B)** Positive expression percentage of ICAM-1, CD11b, B220, and CCR7 was measured in DCs (CD11c^+^F4/80^−^) by flow cytometric analysis. *p < 0.05, **p < 0.01, ***p < 0.001.

Subsequently, we observed the effect of anti-HMGB1 and rHMGB1 on phenotypic and functional changes in the DCs of lung tissue from the LPS-induced ALI mice model. While detecting intercellular adhesion molecule 1 (ICAM-1), CD11b (a marker for myeloid dendritic cells [mDCs]), B220 (a marker of plasmacytoid dendritic cells [pDCs]), and chemotactic factor CCR7 in the surface of lung DCs by flow cytometric analysis, we found that the percentage of ICAM-1, CD11b, or CCR7-positive expression was significantly elevated in the LPS-administrated ALI group in contrast to the control. The increase in ICAM-1, CD11b, and CCR7 percentage was significantly higher in the rHMGB1-treated ALI group relative to those of the ALI group. In addition, the increase in ICAM-1, CD11b, and CCR7 was reduced after anti-HMGB1 treatment (Figures 3A,B). However, the percentage of B220-positive expression among different groups was not significantly different (Figures 3A,B). These data demonstrated that HMGB1 upregulated the percentage of mDCs and enhanced the adhesion and chemotaxis of DCs.

### Inhibition of PI3K/Akt/mTOR Signaling Pathway Weakens the Maturation of DCs *in vivo* and *in vitro*

**Figure 4:**
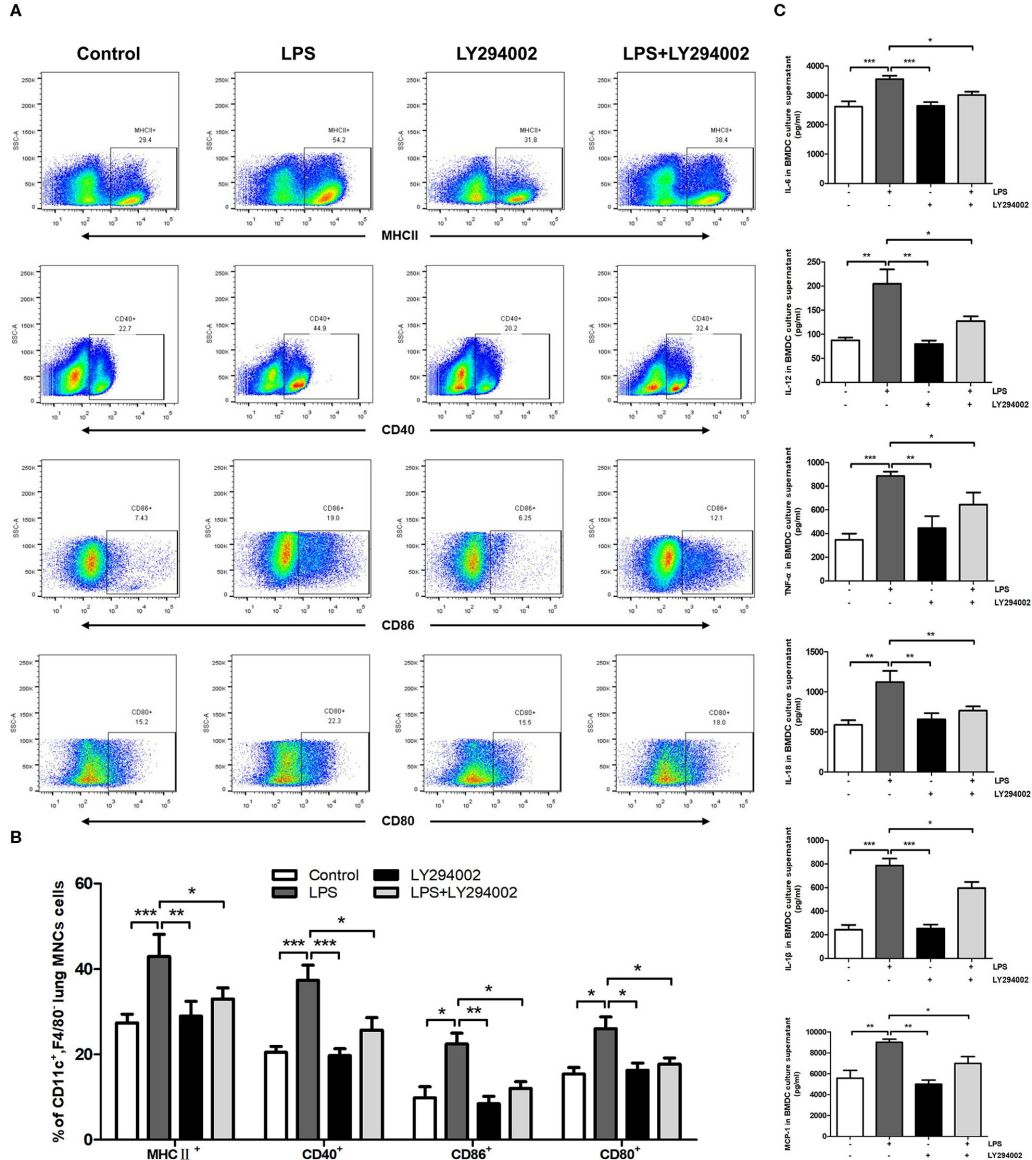
Inhibition of PI3K by LY294002 regulated the maturation of DCs *in vivo* and *in vitro*. **(A,B)** Positive expression percentage of MHCII, CD80, CD86, and CD40 was measured in DCs (CD11c^+^F4/80^−^) by flow cytometric analysis. **(C)** Levels of secreted cytokines TNF-α, IL-6, IL-18, IL-1β, MCP-1, and IL-12 in BMDCs culture supernatant were measured by ELISA. *p < 0.05, **p < 0.01, ***p < 0.001.

We next verified whether the PI3K/Akt/mTOR signaling pathway affects the maturation and function of DCs in the presence of a PI3K inhibitor (LY294002) *in vivo* and *in vitro*. Flow cytometric analysis *in vivo* showed that the percentage of MHCII^+^, CD80^+^, CD86^+^, or CD40^+^ DCs was significantly higher in ALI mice compared with control mice. A significant decrease was also observed in the percentage of MHCII^+^, CD80^+^, CD86^+^, and CD40^+^ DCs in the LY294002-treated mice compared with that in the LPS-induced ALI mice (Figures 4A,B). *In vitro* experiments with BMDCs revealed that stimulation with LPS significantly augmented the levels of TNF-α, IL-6, IL-18, IL-1β, MCP-1, and IL-12 in the culture supernatant. The levels of these proinflammatory cytokines produced by BMDCs were reduced with the inhibition of PI3K (in the LPS+LY294002 group) (Figure 4C). Thus, inhibiting the PI3K/Akt/mTOR signaling pathway attenuated the mature differentiation of DCs in ALI.

### PI3K/Akt/mTOR Signaling Pathway Affected the Phenotypic Changes of DCs in ALI Mice Model

**Figure 5:**
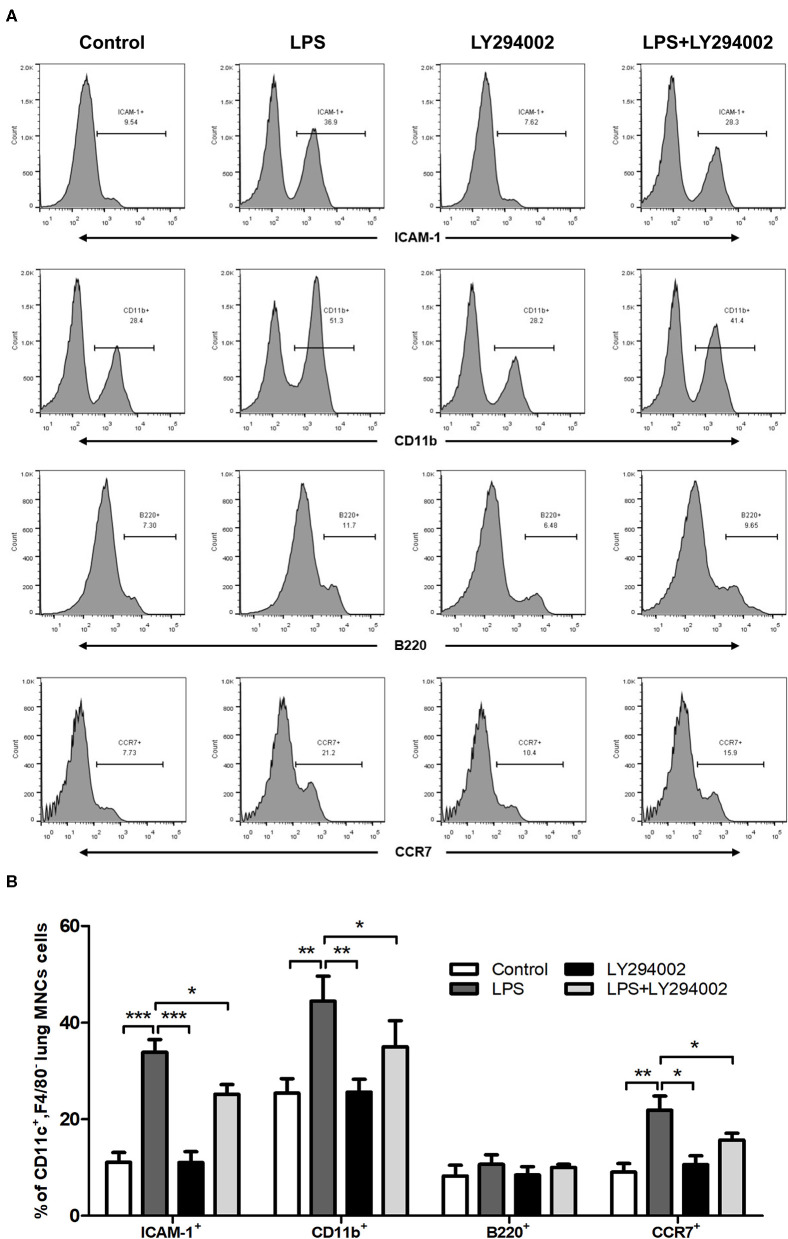
Inhibition of PI3K by LY294002 affected the phenotype and function of DCs. **(A,B)** Positive expression percentage of ICAM-1, CD11b, B220, and CCR7 was measured in DCs (CD11c^+^F4/80^−^) by flow cytometric analysis. *p < 0.05, **p < 0.01, ***p < 0.001.

To demonstrate the role of the PI3K/Akt/mTOR signaling pathway in phenotypic and functional changes of DCs in the ALI mice model, we also treated LPS-induced mice with LY294002 and detected the percentage of ICAM-1, CD11b, B220, and CCR7 in lung DCs by flow cytometric analysis. In comparison with the control group, LPS-induced ALI groups showed a significant increase in the percentage of DCs (CD11c^+^F4/80^−^MNCs) expressing ICAM-1, CD11b, and CCR7. These changes were weakened with LY294002 treatment. In addition, no significant difference arose in the percentage of B220 between different groups (Figures 5A,B). These results suggested that, similarly to HMGB1, the PI3K/Akt/mTOR signaling pathway also influenced the percentage of mDCs and mediated the adhesion and chemotactic ability of DCs to T cells.

## Discussion

In the current study, we show that HMGB1 activates the PI3K/Akt/mTOR signaling pathway in BMDCs and upregulates the expression of PI3K, Akt, and mTOR mRNA and corresponding phosphorylated proteins. HMGB1 and the PI3K/Akt/mTOR pathway form the HMGB1-PI3K/Akt/mTOR signaling pathway in lung DCs, and this pathway then induces the maturation and antigen-presenting ability of lung DCs while also mediating the percentage of mDCs and enhancing the adhesion and chemotactic ability of lung DCs.

HMGB1 functions to regulate the innate immune system. Recent studies have shown that as a late proinflammatory cytokine, HMGB1 has a key role in the pathological progress of ALI and regulates the lung inflammatory response (4, 5). HMGB1 is an upstream mediator of TLR2 and TLR4, the main receptors of HMGB1. Together these form the HMGB1-TLR2, TLR4 pathway, which contributes to inflammatory response via multiple mechanisms (6–8). Besides activating the NF-κB and MAPK pathways, recent studies also have demonstrated that HMGB1 regulates the PI3K/Akt/mTOR signaling pathway in myocardial ischemia/reperfusion injury and LPS-induced pulmonary inflammation in ALI models (9, 10, 13, 14). The PI3K/AKT/mTOR signaling pathway has been shown to contribute to the regulation of cell survival during oxidative stress and to participate in the pulmonary inflammatory progression of ALI (11–14). These studies agree with our results for the relationship between HMGB1 and the PI3K/Akt/mTOR pathway in DCs during ALI.

As specialized antigen-presenting cells pivotal for the initial and adaptive immune response, DCs are outpost cells of immune defense in the respiratory system. Under the stimulation of pathogens, DCs are activated and then mature and migrate to lymph nodes (30). These mature and activated DCs upregulate the surface expression of MHCII and diverse costimulatory molecules (CD80, CD86, and CD40) and release inflammatory cytokines, which train and stimulate Th cells to differentiate into various subtypes (28–30). DCs regulate acute lung inflammation and injury in LPS-induced ALI, and mature DCs participate in aggravating acute lung tissue injury and inflammatory response (17, 18).

In humans and mice, DCs have two important subsets: mDCs (CD11c^+^F4/80^−^CD11b^+^) and pDCs (CD11c^+^F4/80^−^B220^+^). Compared with pDCs, the mDC subset displays high expression levels of costimulatory molecules CD80, CD86, CD40, and MHCII and also better stimulate the proliferation and differentiation of T cells (31). This indicates that the maturity of pDCs is inferior to that of mDCs. Moreover, pDCs do not express LPS-specific receptors TLR2 and TLR4 and therefore cannot be stimulated to maturation by LPS (31). In addition, DCs also express an important adhesive molecule on their cell surface, ICAM-1, which mediates adhesion reaction, and release an important chemokine, CCR7, which has the ability to induce directional chemotaxis (32–34). In our present study, we detected the expression of surface markers (CD80, CD86, CD40, MHC II, B220, CD11b, ICAM-1, CCR7) to reflect the effect of the HMGB1/PI3K/Akt/mTOR signaling pathway on the phenotype and function of DCs in ALI. Our results showed that the HMGB1/PI3K/Akt/mTOR signaling pathway induced upregulation of markers CD80, CD86, CD40, MHC II, CD11b, ICAM-1, and CCR7, suggesting that the proportion of mature DCs increased; the ratio of mDCs also increased, accompanied by the augmentation of antigen presentation, adhesion, and chemotactic ability.

In conclusion, our present study provides evidence of the role of the HMGB1/PI3K/Akt/mTOR signaling pathway at the level of DCs in ALI and further confirms that HMGB1/PI3K/Akt/mTOR signaling participates in the pathological process of ALI by regulating the maturation and functions of DCs.

## Data Availability Statement

The datasets presented in this article are not readily available because access to this dataset is restricted. Requests to access the datasets should be directed to 498676772@qq.com.

## Ethics Statement

The animal study was reviewed and approved by Huazhong University of Science and Technology.

## Author Contributions

RL and YS designed the research. RL, XZ, YY, HZ, PL, SP, and YO performed the experiments. RL analyzed the data and produced the figures. RL, HH, and YS wrote the manuscript. All authors contributed to the article and approved the submitted version.

## Conflict of Interest

The authors declare that the research was conducted in the absence of any commercial or financial relationships that could be construed as a potential conflict of interest.

## Abbreviations

ALI: acute lung injury
LPS: lipopolysaccharide
ARDS: acute respiratory distress syndrome
HMGB1: high mobility group box1
BALF: bronchoalveolar lavage fluid
DCs: dendritic cells
MNCs: lung mononuclear cells
NF-κB: nuclear factor kappa B
TLR: toll-like receptor
BMDCs: bone marrow-derived dendritic cells
MAPK: mitogen-activated protein kinase
cDCs: conventional DCs
RT-qPCR: real-time reverse-transcriptase polymerase chain reaction
ELISA: enzyme-linked immunosorbent assay
IL: interleukin
TNF: tumor necrosis factor
MCP: monocyte chemotactic protein
cDNA: complementary DNA
GAPDH: glyceraldehyde 3-phosphate dehydrogenase
SDS-PAGE: sodium dodecyl sulfate-polyacrylamide gel electrophoresis
PVDF: polyvinylidene fluoride
ICAM: intercellular adhesion molecule
mDCs: myeloid dendritic cells
pDCs: plasmacytoid dendritic cells.
